# Clinical presentation and outcome of enteric fever in adult patients with cancer: a perspective from Pakistan

**DOI:** 10.1099/acmi.0.000719.v3

**Published:** 2024-05-14

**Authors:** Seemal Aslam, Salma Abbas, Summiya Nizamuddin, Muhammad Shehbaz, Azra Parveen, Faisal Sultan, Aun Raza

**Affiliations:** 1 Fellow Infectious Diseases, Department of Internal Medicine, Shaukat Khanum Memorial Cancer Hospital and Research Centre, Lahore, Pakistan; 2 Consultant Internal Medicine & Infectious Diseases, Shaukat Khanum Memorial Cancer Hospital and Research Centre, Lahore, Pakistan; 3 Consultant Medical Microbiologist, Shaukat Khanum Memorial Cancer Hospital and Research Centre, Lahore, Pakistan

**Keywords:** cancer, enteric fever, immunocompromised, *Salmonella typhi*, *Salmonella paratyphi*, typhoid

## Abstract

**Introduction.** Enteric fever is a significant health concern in endemic countries. While extensive research has been conducted to understand its presentation and outcomes in non-cancer patients, limited data exist on its impact on cancer patients. This descriptive study aims to investigate the clinical presentation and outcome in cancer patients.

**Methodology.** This retrospective observational study analysed 90 adult cancer patients from a single centre in Pakistan from January 2017 to December 2022. Inclusion criteria involved documented blood culture infections with *Salmonella typhi* or *paratyphi* A, B, or C. We examined clinical presentation, laboratory parameters, antimicrobial resistance, complications, and outcomes. Additionally, we explored the effects of chemotherapy, comorbidities, type of malignancy, and patient age on complications and mortality.

**Results.***Salmonella typhi* was the most prevalent organism (72.2 %), followed by *Salmonella paratyphi* A (22.2 %) and B (5.5 %). Variably-resistant isolates constituted 51.5 %, multi-drug resistant (MDR) isolates accounted for 20 %, extensively drug-resistant (XDR) for 14.4 % and ESBL-producers for 15.5 %, of all enteric fever infections. Enteric fever-associated complications were observed in 21.1 % of cases. Chemotherapy in the preceding month did not affect mortality, nor did age, gender, or malignancy type. However, comorbidities were statistically significant for mortality (*p*-value 0.03). A total of 8.8 % of patients required ICU care, and the all-cause 30 day mortality rate was 13.3 %

**Conclusion.** Enteric fever remains prevalent in our geographical region. Unlike non-typhoidal *Salmonella* (NTS), enteric fever does not behave differently in an immunocompromised population, including cancer patients.

## Data Summary

All supporting data is present in the manuscript.

## Introduction

Enteric fever is an acute febrile illness caused by a Gram-negative bacterium belonging to the genus Salmonella. It includes serovar *Salmonella typhi,* and *paratyphi A, B* and *C*. Its distribution is worldwide with the highest disease burden and endemicity in middle- and low-income countries in Asia, Africa and the Middle East [1]. It is a food and water-borne infection with a faecal–oral route as a primary transmission mode.

The incidence of enteric fever has decreased in industrialized countries; however, it remains endemic in low socioeconomic countries. According to a report by WHO, the estimated number of cases of enteric fever in 2019 was 9.2 million and 1,10,000 deaths attributable to this disease [2]. Moreover, the incidence of multi-drug resistance (MDR) and extensively drug resistance (XDR) cases are also on the rise due to unnecessary use of antibiotics and lack of antibiotic stewardship programmes, especially in our region [3]. The clinical presentation of enteric fever is variable. Fever is the most predominant feature. Other symptoms include constipation or diarrhoea, headache, dry cough, abdominal pain, malaise, anorexia and vomiting.

Regarding the clinical course of salmonella infections in immunocompromised patients, non-typhoidal salmonella (NTS) are known to cause more invasive infections in this population and this has been studied extensively [4,5]. However, limited data suggests that typhoidal illness does not depend on host factors [4]. Cancer alters our immune system by various mechanisms. Moreover, treatment modalities like chemotherapies and bone marrow transplantations, used for the treatment of cancer, work by suppressing host immune response in various ways. This leads to a higher incidence of infections with more significant morbidity and mortality. There is minimal data regarding enteric fever in cancer patients and other immunocompromising conditions like HIV, autoimmune diseases, patients on immunosuppressive medication, or diabetes. Khan *et al*. reported ten cases of typhoid fever with HIV and compared it with 32 immunocompetent controls in Durban, South Africa, and found no difference in epidemiology and clinical characteristics [6]. Another study conducted in Cambodia included 254 patients, of which 7.8 % (*n*=20) were immunosuppressed, including one cancer patient. There was no difference in immunocompetent or immunosuppressed patients in terms of clinical presentation or complications [7]. In another retrospective study conducted on 134 patients with salmonella infections, including 34 % diabetic patients and 5.2 % with underlying malignancy, not a single patient died of *Salmonella typhi,* while overall mortality of 5.2 % was only seen in NTS infection [8]. Although in a case series of 12 systemic lupus erythematosus (SLE) patients, with 11 patients on high-dose steroids, septic arthritis was seen more commonly followed by sepsis syndrome and acute respiratory distress syndrome. However, there was no impact on mortality [9].

There is limited data on typhoidal illnesses in the cancer population and the aim of this study is to explore the clinical presentation and outcome of enteric fever in cancer patients.

## Methods

**Studydesign**: It was a retrospective observational study, which was conducted in Shaukat Khanum Memorial Cancer Hospital and Research Centre (SKMCH and RC), Lahore, Pakistan.

**Study Period**: 1 January 2017, to 31 December 2022.

**Datacollection**: Data collection was done retrospectively using a preformed questionnaire using electronic health record system.

**Study population**:

- **Inclusion criteria**

1. Adult individuals aged 18 years or older.
2. Diagnosis of malignancy, whether of solid organ or haematological origin.
3. Culture proven bloodstream infection with *Salmonella typhi* or *Salmonella paratyphi* A, B, or C.
4. Receipt of treatment at SKMCH and RC, Pakistan, in-patient or out-patient.

- **Exclusion criteria**

1. Patients with age <18 years.
2. Non-cancer patients

**Operational definitions:**

**Enteric fever** was defined as a positive blood culture with *Salmonella typhi*, *Salmonella Paratyphi* A, B, or C.

**Fever** was defined as body temperature >38 °C checked with a thermometer.

**Generalized weakness** was considered as fatigue or malaise, which compromised patient wellbeing and it was recorded subjectively.

**Hepatitis** was defined as two or more times increase in liver transaminases during this illness [10].

**Acute kidney injury** was defined as rise of serum creatinine for ≥0.3 mg dl^−1^ in 48 h or 1.5 times from baseline in 7 days [11].

**Haemodynamic instability** was defined as systolic blood pressure <90 mmHg and/or diastolic <60 mmHg or mean arterial pressure (MAP) below 65 mmHg.

**Encephalopathy** was defined as any neurological manifestation like confusion, delirium, stupor, psychosis, meningism, seizures or GCS<15 without alternative reason [12].

**Variably-resistant infection** was defined as isolated sensitive to first-line antibiotics (ampicillin, trimethoprim-sulfamethoxazole, and chloramphenicol) and sensitive to third-generation cephalosporin with or without resistance to second-line drugs (fluoroquinolones).

**Multi-drug resistant (MDR) infection** was defined as those isolates resistant to first-line antibiotics, but sensitive to third-generation cephalosporins with or without resistance to second-line drugs.

**Extensively drug-resistant (XDR) infection** was defined as resistance to first-, second-line antibiotics and third-generation cephalosporins.

**ESBL-producing organisms** were considered resistant to third-generation cephalosporin but may be sensitive to chloramphenicol, co-trimoxazole or fluoroquinolones.

### Data analysis

In this study, we conducted a comprehensive analysis of patient data, which encompassed demographics, clinical presentations, laboratory parameters, complications, and outcomes. The data collected was entered and analysed using IBM SPSS Statistics Version 20. Descriptive and analytical statistics were performed. Cross-tabulation and chi-square testing was used to find statistical significance between age, gender, type of malignancy, presence of comorbidities and chemotherapy with complications and mortality considering *P*<0.05 statistically significant.

## Results

We collected data from 90 cancer patients who had documented blood culture positive results for *Salmonella typhi* or *paratyphi* A, B or C. Female to male ratio was 8 : 7. Mean age of the participants was 36.79±16.1 years. Out of 90 patients, 65.5 % (*n*=59) had solid organ malignancy while 34.4 % (*n*=31) suffered from haematological malignancy. Among solid organ malignancies, breast (*n*=14) and gastrointestinal (*n*=14) were the most predominant malignancies, while in haematological malignancies, Hodgkin’s lymphoma (*n*=9) predominated. Demographics and baseline characteristic of the patients are shown in Table 1.

**Table 1.** Baseline clinical characteristics and outcome of the patients

Characteristics	*n* (%)	Overall complications with *P*-value		Overall mortality with *P*-value	
**Age (years)** 18–59	78 (86.6 %)	14 (17.9 %)	0.12	9 (11.5 %)	0.19
Above 60	12 (13.3 %)	5 (41.6 %)		3 (25 %)	
**Gender** Male	42 (46.6 %)	9 (21.4 %)	1.0	5 (11.9 %)	0.76
Female	48 (53.3 %)	10 (20.8 %)		7 (14.5 %)	
**Primary diagnosis**					
Solid organ	59 (65.5 %)				
GI	14				
Breast	14				
Lung	4				
Brain	7	16 (27.1 %)		10 (16.9 %)	
Genitourinary track	11				
Musculoskeletal	6				
Endocrine	3		0.06		0.2
Haematological	31 (34.4 %)				
HL	9				
NHL	2				
ALL	4				
AML	1				
CLL	4				
CML	6				
DLBCL	4	3 (9.6 %)		2 (6.4 %)	
MM	1				
**Comorbidities**					
DM	5 (5.6 %)				
IHD	3 (3.3 %)	4 (36.4 %)	0.23	4 (36.4 %)	0.037
HTN	2 (2.2 %)				
COPD	1 (1.1 %)				
**Chemotherapy (within4 weeks)**					
Yes	22 (24.4 %)	4 (18.2 %)	1.0	3 (13.6 %)	0.6
No	68 (75.5 %)				
**Clinical presentation**					
Fever	82 (91.1 %)				
Generalised	33 (36.7 %)				
weakness/malaise	27 (30 %)				
Nausea/vomiting	27 (30 %)				
Diarrhoea	27 (30 %)				
Cough	22 (24.4 %)				
Abdominal pain	13 (14.4 %)				
Headache	7 (7.8 %)				
Anorexia	6 (6.7 %)				
Constipation	5 (5.6 %)				
Flu/sore throat	1 (1.1 %)				
Seizure					
Encephalopathy	9 (10 %)				
AKI	7 (7.8 %)				
Hepatitis	5 (5.6 %)				
Intestinal perforation	1 (1.1 %)				
**CU transfer**					
Yes	8 (8.8 %)				
No	82 (91.1 %)				
**Outcome4 weeks**					
Alive	78 (86.6 %)				
Dead	12 (13.3 %)				

**Fig. 1.**
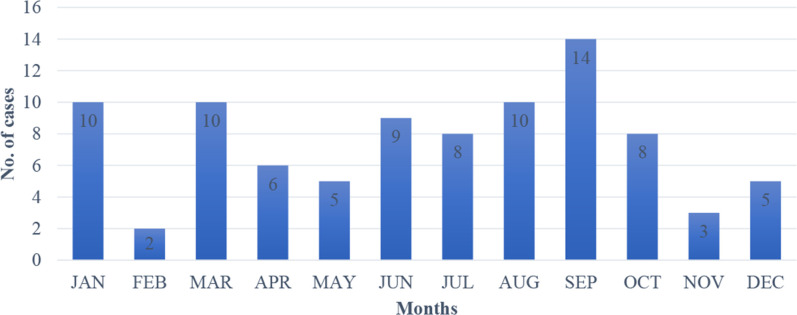
Month-wise distribution of cases: a 5 year analysis.

**Fig. 2.**
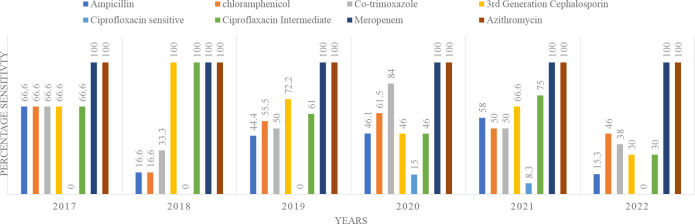
Percentage *Salmonella typhi* sensitivity to antimicrobials: a temporal trend.

Fever was the most common clinical presentation 91.1 % (*n*=82), followed by generalized weakness, nausea/vomiting, diarrhoea and cough (Table 1). Month-wise distribution of cases is shown in Fig. 1. Blood cultures and subgroup frequencies are shown in Table 2. Antibiotic resistance of *Salmonella typhi* over a period 5 years is shown in Fig. 2.

**Table 2.** Blood cultures and subgroup frequencies

Organisms	Variably-resistant	MDR	XDR	ESBL	Total
*Salmonella typhi*	26	13	14	12	**65** (72.2 %)
*Salmonella paratyphi A*	18	2	0	0	**20** (22.2 %)
*Salmonella paratyphi B*	2	3	0	0	**5** (5.5 %)
**Total *n*** (**%**)	**46** (51.5 %)	**18** (20 %)	**14** (14.4 %)	**12** (15.5 %)	**90** (100 %)

CRP was checked in only 36.6 % (*n*=33), and mean CRP was 162.6±152.4. Mean haemoglobin was 10.9±2.68 g dl^−1^. Mean leucocyte count was 15.1±43.1×10³/ul. A total of 45.6 % (*n*=41) patients received piperacillin-tazobactam empirically, but after the culture results were reported third-generation cephalosporins (58.9 %) were the most frequent antibiotic prescribed (Table 3). Enteric fever associated complications in solid organ malignancy patients were more than haematological malignancies (*P*=0.07) and mortality in this group was also more (*P*=0.14) (Table 1). Among enteric fever related complications, encephalopathy was seen in 10 % (*n*=9), followed by acute kidney injury (AKI) and hepatitis and intestinal perforation (Table 1). Haemodynamic instability was seen in 11.1 % (*n*=10) patients. A total 24.4 % of patients (*n*=22) received chemotherapy 4 weeks prior to developing this infection, and it was not statistically linked with overall enteric fever associated complications (*P*=0.5) and mortality (*P*=0.6), neither did age subgroups, or gender (Table 1). All-cause 30 day mortality was 13.3 % (*n*=12). Percentage of all-cause mortality was 11.9 %(*n*=5) in variably-resistant group, 13 % (*n*=3) in MDR, 7.6 %(*n*=1) in XDR, and 25 % (*n*=3) in ESBL group. Mean length of hospital stay was 5.72±4.79 days.

**Table 3.** Antibiotic use: empiric and targeted

	Before culture report		After culture report	
Antibiotics	Frequency (*n*)	Percent (%)	Frequency (*n*)	Percent (%)
Piperacillin-tazobactam	41	45.6	2	2.2
Cephalosporin	9	10.0	53	58.9
Carbapenem	9	10.0	19	21.1
FQs	14	15.6	2	2.2
Azithromycin	2	2.2	10	11.1
Others	8	8.9	2	2.2
None	7	7.8	2	2.2
Total	90	100.0	90	100.0

## Discussion

Enteric fever is a widespread infectious disease that affects millions of people every year. NTS infections have a clear association with invasive disease in immunocompromised patients, but severe typhoidal illness have not been implicated in immunodeficiency states [4,5]. There is very limited data regarding the clinical manifestations and outcomes of enteric fever in cancer patients. Hence, we add to the existing literature on clinical characteristics of enteric fever and its outcome in cancer patients.

In our study, infection was most frequently caused by *Salmonella typhi* followed by *Salmonella paratyphi* A and B. There was no documented *Salmonella paratyphi* C infection. This is consistent with the non-cancer and immunocompetent population worldwide, as reported by the Centers for Disease Control [13]. In our study, overall, 48.8 % (*n*=44) of blood cultures showed extensive antimicrobial resistance, while 51.1 % (*n*=46) were variably-resistant per our operational definition.

Antimicrobial resistance is on the rise worldwide, possibly due to the injudicious use of antibiotics and lack of stewardship programmes. A systematic analysis of antimicrobial resistance of *Salmonella typhi* from 1972 to 2018 suggested increasing prevalence of MDR strains in Africa along with increases in MDR as well as XDR strains in Asia [14]. In another study conducted in three hospitals of Pakistan, the overall incidence of typhoid fever declined in 2015 as compared to 1992, but subgroup analysis suggested increasing prevalence of MDR and XDR strains [15]. Moreover, there was an outbreak of XDR infections in the Sindh province of Pakistan in 2016 [16] and later, genetically variable strains of this XDR organism spread beyond the originating province [17]. In contrast, our study demonstrated a lower prevalence of MDR and XDR strains. This could be due to the small sample size in our study or dilution of the these isolates in the overall sample due as our hospital has a wide catchment area and caters to patients from various locations in Pakistan.

The most common clinical symptom seen in our patients was fever, followed by general weakness, nausea/vomiting, diarrhoea, cough, abdominal pain, headache, anorexia, constipation and sore throat, while only one patient presented with seizures. This patient had oligodendroglioma, however there was no observed progression of the malignancy in the patient during initial assessment. Lumber puncture was not performed in the said patient due to thrombocytopenia, hence the cause of seizures remained unknown. Clinical presentation of enteric fever among immunocompetent hosts are considerably similar, as per literature review. A prospective analysis of 50 patients with no co-morbidities done in Mumbai revealed similar presenting complaints [18].

Haemodynamic instability was seen in 11.1 % of the patients. Only 20 % of these patients presented with diarrhoea. The frequency of haemodynamic instability increased with increasing antimicrobial resistance, with highest prevalence in XDR and ESBLs. This result is consistent with the trend seen in a surveillance study previously conducted, in which haemodynamic instability and other complications were more commonly associated with XDR infections, but the population in this study was non-immunocompromised unlike our study [19].

In our study cohort, 10 % of the patient had altered sensorium. Out of these, 33.3 % (*n*=3) had primary brain malignancy, while in 44 % of patients, the cause for this presentation was attributed to sepsis and multiorgan failure. Neuropsychiatric manifestation of enteric fever usually occurs when the patient has severe sepsis and it can be seen in 5–35  % of the cases [20]. Our patients who developed these neuropsychiatric complications were critically ill and that can be seen with high mortality in this cohort. Acute kidney injury (AKI) was seen in 7.8 % (*n*=7) patients, out of which 28.5 % had diarrhoea, and cause of AKI was attributed to fluid loss. While in the remaining patients, sepsis was considered to be the cause of AKI. Enteric fever can potentially affect kidneys by a variety of mechanisms. Enteric fever has been reported to cause pyelonephritis and glomerulonephritis [21], however none of our patients developed these complications. In rare cases, enteric fever can lead to AKI by causing rhabdomyolysis [22]. One patient, who underwent three phase esophagostomy developed concealed intestinal perforation post-operatively, he was managed conservatively. Intestinal perforation is one of the most dreadful complications of enteric fever and it is reported between 0.7–7.6 % of cases [23]. Its pathophysiology is linked to inflammation of Peyer’s patches and it can involve any part of the gut from duodenum to colon [24]. Most of our patients had carcinoma of the gastrointestinal tract, yet intestinal perforation was reported in just one patient, who underwent esophagostomy. It implies that gastrointestinal malignancy does not predispose patients to develop intestinal perforation with enteric fever. Amongst those who developed hepatitis, none had liver involvement with primary malignancy and their baseline liver function tests were normal. Resolution of transaminitis occurred once enteric fever was treated.

Chemotherapy was received by 24.4 % of the patients. Although chemotherapy leads to many gut-related complications including neutropenic colitis and predisposes to infections [25], yet complications and mortality outcomes were no different in chemotherapy vs non-chemotherapy groups. It implies that immunosuppressed status of the patient is not associated with the worse outcomes. Similarly, subgroup analysis of age, gender or type of malignancy showed no statistically significant impact on development of complications or on mortality. However, mortality was higher in patients with comorbidities (*P*=0.03). Overall, the fatality rate in our study was 13.3 %, which is higher compared to other studies. A surveillance study conducted in Nepal, Bangladesh and Pakistan for the year 2016–19 estimates it to be 0.07 % [26]. Patient immunological status or comorbidities were not studied in that analysis. The major limitation in our study was that it did not segregate the cause of death as enteric vs all other causes and we suggest more research to explore this further.

Laboratory parameters including total leucocyte count and haemoglobin cannot be used as estimation of severity of enteric fever due to presence of haematological malignancies and treatment modalities like chemotherapies which cancer patients receive. Hence characteristic lymphopenia that can be seen in non-cancer patients cannot be reliably implemented in this group of patients.

Enteric fever is most commonly seen during summers and rainy seasons, May through October, owing to increased transmission [27]. We also observed a similar trend in our study.

Our study has several limitations. This is a retrospective observational single-centre study, with a small sample size. Hence our results cannot be extrapolated to a broader population with patients from different backgrounds. Our study lacked controls. Moreover, the reasons for high mortality and the exact cause of death for each patient was not explored.

The major strength of our study is that it included only cancer patients and adds to the existing, scarce literature on the characteristics of enteric fever in this population. While the results of our study cannot be generalized to the general population, it provides useful insights into the epidemiology of enteric fever in cancer patients, its clinical manifestations including complications and death. We recommend that further studies be performed to further explore the characteristics of enteric fever in this population.

## Conclusion

In conclusion, our study sheds light on the clinical presentation and outcomes of enteric fever in cancer patients. Clinical presentations and the frequency of complications among cancer patients were similar to those observed in the general population. Chemotherapy did not seem to significantly impact the outcome. Nevertheless, the overall high all-cause mortality rate, warrants further studies to fully elucidate this.

## Abbreviations

CRP: c-reactive protein
ESBL: extended spectrum beta-lactamase
MDR: multi-drug resistant
NTS: non-typhoidal *Salmonella*
XDR: extensively drug resistant
